# Novel Nanocomposite Hydrogels Based on Crosslinked Microbial Polysaccharide as Potential Bioactive Wound Dressings

**DOI:** 10.3390/ma16030982

**Published:** 2023-01-20

**Authors:** Maria Minodora Marin, Madalina Albu Kaya, Durmus Alpaslan Kaya, Roxana Constantinescu, Bogdan Trica, Ioana Catalina Gifu, Elvira Alexandrescu, Cristina Lavinia Nistor, Rebeca Leu Alexa, Raluca Ianchis

**Affiliations:** 1 National Research and Development Institute for Textile and Leather, Division Leather and Footwear Research Institute, Department of Collagen, 93 Ion Minulescu Str., 031215 Bucharest, Romania; 2 Advanced Polymer Materials Group, Faculty of Applied Chemistry and Materials Science, Politehnica University of Bucharest, 1–7 Polizu Street, 01106 Bucharest, Romania; 3 Faculty of Agriculture, Hatay Mustafa Kemal University, Hatay 31060, Turkey; 4 National Research and Development Institute for Chemistry and Petrochemistry ICECHIM, Spl. Independentei 202, 6th District, 0600021 Bucharest, Romania

**Keywords:** hydrogels, antimicrobial, polysaccharide, salecan, silver nanoparticles

## Abstract

A multitude of dressings have been developed to promote wound repair, such as membranes, foams, hydrocolloids and hydrogels. In this study, a crosslinked polysaccharide hydrogel was mixed with a bioactive ingredient to synthesize a novel nanocomposite material to be used in wound healing. Variation of the ratio between hydrogel components was followed and its effect was analyzed in regard to swelling, degradation rate and thermo-mechanical behavior. The resulting crosslinked structures were characterized by FTIR and microscopy analyses. The antimicrobial activity of the crosslinked hydrogels loaded with bioactive agent was evaluated using two bacterial strains (Gram-positive Staphylococcus aureus and Gram-negative bacteria Escherichia Coli). All the results showed that the new synthesized biopolymer nanocomposites have adequate properties to be used as antibacterial wound dressings.

## 1. Introduction

Patients with serious burn wounds need specialized care to reduce trauma and mortality [1]. The main factors contributing to morbidity and mortality are sepsis and wound infection [2]. The type and number of bacteria that colonize the burn wound affect invasive wound infection [1]. Normal wound dressings, such as gauzes and bandages, are inadequate for this trauma, especially for more severe wounds, due to a number of drawbacks such as poor absorption, frequent replacement requirements, high adhesiveness, and sticking to the wound tissue, which could harm the newly formed epidermis [3]. This area of biomaterial science is rapidly advancing, with an increased emphasis on creating innovative wound dressing formulations to fight multidrug-resistant bacteria [4]. Hemostasis, inflammation, epithelial cell migration and proliferation, followed by epithelial tissue remodeling and wound healing, constitute the complicated process of wound healing [5]. The hemostasis and inflammatory stages, which often last between 24 h and three days, constitute the organism’s initial line of defense against bacterial infection [5]. It is ideal for a wound dressing to contain an active antibacterial component that should inhibit biofilm formation in the first 24–48 h since it is crucial to prevent bacterial migration and attachment to the wound during this early phase [6]. Hydrogels are perceived as promising materials in wound care applications. Hydrogels facilitate an intrinsic hydrated environment which allows the damaged tissue to regenerate by aiding autolytic debridement [7,8]. The dressings offer a temporary barrier against superficial infections, speeding up the healing process [9]. An effective dressing for treating wounds must exhibit the following features: excellent biocompatibility, adequate swelling behavior to maintain the wound’s moist environment, good mechanical properties in the wet state and cell adhesion capacity [10,11]. Natural or synthetic materials of many types have been used for skin care [12]. In particular, hydrogels used for damaged skin have drawn a lot of interest because, in contrast to other materials, they are uniquely able to mimic the natural skin microenvironment [13]. A hydrogel represents a three-dimensional network of interlaced hydrophilic polymers with a high-water up-take rate and lots of holes. Since the majority of hydrogels are biocompatible, can be controlled and hold several sorts of medicinal substances, hydrogels are frequently employed in medical applications [14,15]. Due to their exceptional qualities such as biodegradability, renewability, biocompatibility and ease of chemical manipulation, polysaccharides-based hydrogels have been the subject of ongoing study for medical applications [16]. Monosaccharide units connected by glycosidic bonds form the long chains of carbohydrates known as polysaccharides [17]. Natural polysaccharides include microbial polysaccharides, which can be produced using bacterial or fungal cultures from renewable biomasses such as algae or plants [18]. Microbial polysaccharides have applications in different industrial sectors such as pharmaceutical and cosmetics, oil extraction, food, wastewater treatment and medical applications. In the past few years, the most used microbial polysaccharides were xanthan gum, gellan, dextran, pullulan and curdlan [18,19,20].

Due to its superior hydrophilicity and biocompatibility, the new microbial polysaccharide-salecan can be used to create hydrogel for biomedical purposes [21]. Additionally, salecan has a lot of functional hydroxyl groups on its chains, making it simple to modify chemically [7,22]. Few hydrogels made entirely of salecan have, however, been synthesized, a fact that is intimately tied to the behavior of the biopolymer since it lacks the ability to form hydrogels without structural modification. Thus, H bonds were generated when salecan was physically crosslinked through heat treatment [23] or the use of graphene oxide sheets [24]. One study claimed that salecan was ionically crosslinked using chromium ions [25], but in these instances, weak hydrogels were still obtained [23]. In a recent study, salecan was covalently crosslinked with citric acid, a green crosslinker, and good wet-environment stability was attained for a variety of biopolymer and citric acid formulations. Additionally, the potential for using some salecan hydrogel formulations in additive manufacturing process was explored [26].

Silver nanoparticles have extremely strong antibacterial activity against a wide range of bacteria, including those that are multidrug resistant and found in hospitals [27,28]. Silver nanoparticles are a fantastic candidate for the antibacterial component of wound dressings due to their complex antibacterial mechanism. Unlike antibiotics, silver nanoparticles are exceedingly difficult for bacteria to become resistant to [29]. Silver nanoparticles also offer mechanical characteristics and are non-toxic. They are made to provide a moist environment around the wound and are able to promote continuous oxygen during the healing process. The silver nanoparticles in the material also work to stop infection and microbial growth around the wound site [30]. The antimicrobial properties of silver, which are used in topical preparations such as dressings, creams and ointments to apply on wounds, are now well known, and it seems obvious that, over the past few years, a great interest in silver and, in particular, silver nanoparticles and their applications for wound healing purposes has been growing [31,32]. Additionally, biocompatible materials based on silver nanoparticles are available, affordable and easy to apply and remove from the wound location owing to the accessibility of natural resources for materials [32].

The objective of this study was to create novel salecan-based nanocomposite hydrogels with increased antibacterial activity and mechanical stability in wet environments. Thus, silver nanoparticles were added to the biopolymeric matrix to create stable hydrogels that may be used as antibacterial wound dressings. In addition to extending the use of microbial polysaccharide crosslinked hydrogels as bioactive wound dressings, this study is the first to examine crosslinked salecan hydrogels that have been compounded with silver nanoparticles. The morphological, thermal, physical and mechanical characteristics of the obtained hydrogels were studied to investigate their practical usefulness as dressings. Additionally, the antibacterial activity against Gram-positive Staphylococcus aureus and Gram-negative Escherichia coli was evaluated.

## 2. Materials and Methods

### 2.1. Materials and Preparation Method

Microbial polysaccharide-salecan was purchased from Suzhou Chemicals (Suzhou, China), Citric acid from SC Remed Prodimpex SRL (Bucharest, Romania). Silver nanoparticles were prepared in our laboratory using the method described by Z. Wu et al. [33]. Ultrapure water and solutions with different pH values (pH = 2; 7.4; 11) were prepared in our laboratory using HCl 37% and NaOH from SC Chimreactiv SRL (Bucharest, Romania) and Na_2_HPO_4_ from Reactivul (Bucharest, Romania). Phosphate buffered saline was purchased from Sigma (St. Luis, United States and contains NaCl-0.138M; KCl—0.0027M.

Sulphuric acid (95–97% purity) from Supelco (Darmstadt, Germany) and phenol (min. 99.5% purity) from SC Chimreactiv SRL (Bucharest, Romania) were used.

The new polysaccharide crosslinked hydrogels and their silver nanocomposites were obtained according to the composition from Table 1.

**Figure 1:**
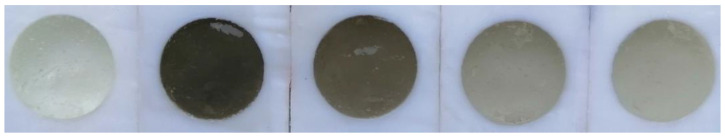
The initial hydrogel samples.

Firstly, we prepared a stock solution of 5% *w/v* citric acid (CA) and silver nanoparticles in deionized water. The dispersion was magnetically stirred at 600 rpm for 24 h and then ultrasonicated 5 min in an ice bath. Salecan polysaccharide (S) was added on the sample specific silver dispersions. The obtained mixtures were kept at 40 °C for 20 h and the resulting hydrated biopolymer hydrogels were placed in cylindrical molds with 0.6 mm high (Figure 1). All the samples were freeze dried and exposed to a heat treatment for 24 h at 50 °C followed by thermal shock for 40 min at 120 °C. The obtained crosslinked hydrogels were used for further characterization.

**Figure 2:**
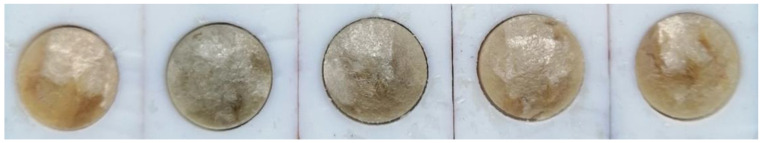
The final samples resulted after freeze drying and thermal treatment.

The final samples resulted after being freeze dried and the thermal treatment can be observed in Figure 2.

### 2.2. Analytical Methods

#### 2.2.1. Fourier Transform Infrared Spectrometry (FT-IR)

Microbial polysaccharide crosslinked hydrogels were structurally characterized in powder form using Fourier Transform Infrared Spectrometry—ATR mode. The tests were achieved on a Tensor 37 Bruker Fourier transform infrared spectrophotometer. All the FT-IR spectra were obtained in the 4000–400 cm^−1^ wavelength range.

#### 2.2.2. Scanning Electron Microscopy (SEM)

Environmental scanning electron microscopy (ESEM-FEI Quanta 200, Eindhoven, The Netherlands) was employed to analyze the internal structure and morphology of the printed freeze-dried hydrogels. The samples were not sputter-coated; they were examined in their natural state.

#### 2.2.3. Transmission Electron Microscopy (TEM)

Transmission electron microscopy (TEM) tests were performed on the freeze-dried hydrogels. All the samples were evaluated using BF-TEM (Bright Field Transmission Electron Microscopy) mode at an accelerating voltage of 200 kV on a TECNAI F20 G² TWIN Cryo-TEM (FEI, Hillsboro, OR, USA). EDX spectra were obtained using an X-MaxN 80T detector (Oxford Instruments, Abingdon, UK).

#### 2.2.4. Thermogravimetric Analysis (TGA)

The variations of the denaturation temperature after the addition of the Ag nanoparticles were analyzed by thermogravimetric analysis (TGA). All the tests were accomplished using a NETZSCH TG 209 F1 Libra instrument (Selb, Germany) with a flow rate of nitrogen of about 20 mL/min, scanning from 30 to 700 °C and at a heating rate of 10 °C/min). Every sample with a mass between 3.5 and 4.5 mg was introduced in aluminum pots before examination.

#### 2.2.5. Determination of the Crosslinking Degree and the Behavior of Crosslinked Hydrogels in Wet Conditions

The part of the biopolymeric mixture converted to the crosslinked form was determined for all the samples using the phenol-sulfuric acid method [22,25]. The crosslinking degree, CD, was calculated using Equation (1), where *mi* represents the mass of salecan in each sample and *mf* represents the mass of crosslinked salecan.

CD salecan % = *mf* salecan × 100/*mi* salecan (1)

For swelling degree determinations, we obtained samples with the same compositions in cubical silicon molds. The swelling degree of the crosslinked samples was evaluated by incubation in 3 solutions with different pHs (2; 7.4; 11) and a temperature of 37 °C. The swelling degree was as definite as the ratio of weight increase (w–w0) to the initial weight (w0). Each obtained hydrogel was analyzed in duplicate for the three pH values.

#### 2.2.6. Mechanical tests 

Using a DMA Q800 (TA Instruments, New Castle, DE, USA), dynamic mechanical examination of the samples was taken. Round sponge samples of 15 mm in diameter and 10 mm in thickness were used for the measurements, which were analyzed in compression mode at 37 °C. All the equilibrium-swelled hydrogels were compressed with a ramp force of 0.01 N/min, from 0.01 to 1.5 N. The method used to evaluate the hydrogel samples was the compression modulus.

#### 2.2.7. Antimicrobial Activity

The antimicrobial assay was performed using standard strains from the ICPI, Microbiology Department Collection, as follows: *Escherichia coli* ATCC 11229, a Gram-negative strain, and *Staphylococcus aureus* ATCC 25923, a Gram-positive bacterial strain. The qualitative screening of the antimicrobial properties was performed by an adapted spot diffusion method. The protocol of this method was described in our previous research work [34]. Bacterial and yeast suspensions of 1 × 108 CFU_mL^−1^ (corresponding with 0.5 McFarland standard density) obtained from 24–48 h microbial cultures developed on Muller Hinton agar (MHA). The plates were left at room temperature to ensure the equal diffusion of the compound in the medium and then incubated at 37 °C for 24–48 h. After 24 h, the inhibition zone diameters of the samples were measured. All the measurements were achieved in triplicate.

## 3. Results and Discussions

### 3.1. Morpho-Structural Changes of the Salecan-Based Nanocomposites

**Figure 3:**
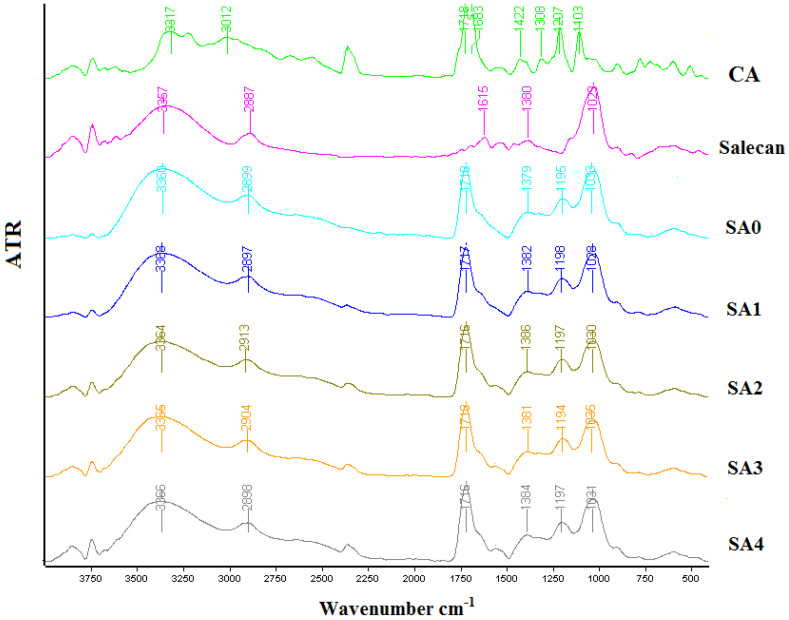
The FT-IR spectra of the obtained crosslinked hydrogels, salecan and citric acid.

Infrared spectrometry was used to determine the novel hydrogels structures, which are shown in Figure 3. The salecan/silver nanoparticle biomaterials spectra revealed the distinctive infrared bands of their components. Thus, salecan FTIR spectra depicted its characteristic peaks at 3360 cm^−1^ due to OH stretching vibration, ~2800 cm^−1^ due to asymmetric stretching vibration of CH_2_ groups and 1029 cm^−1^ corresponding to the OH vibration of the glucopyranose ring [35,36]. The FTIR spectra of citric acid displays several signals at 3316 and 3312 cm^−1^ peaks associated with stretching vibration of OH, 1681–1718 cm^−1^ a split peak corresponding to C=O stretching vibration from COOH groups, 1200–1300 cm^−1^ peaks attributed to C-O-C bonds and 1103 cm^−1^ for C-OH [37,38,39]. The crosslinked samples exhibited some changes in the wavelength’s values of both individual components, corresponding to the OH and COOH peaks. These abatements are associated with covalent crosslinking between salecan and citric acid as a result of the esterification reaction [40]. However, we did not identify significant modifications as a function of silver nanoparticles addition. For instance, with increasing silver nanoparticles concentration, the peak corresponding to the OH stretching registered for salecan crosslinked sample at 3360 cm^−1^ ranged between 3364 cm^−1^ and 3368 cm^−1^ in the nanocomposite samples, the highest value corresponding to the richest sample in silver nanoparticles.

**Figure 4:**
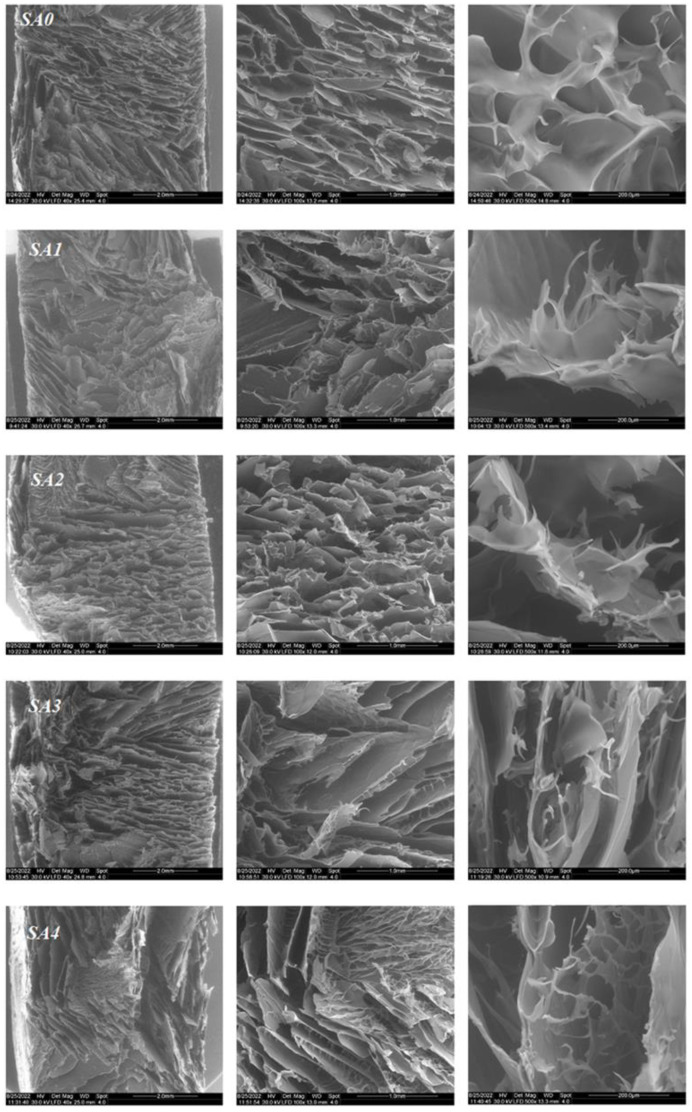
SEM images of the obtained lyophilized samples (×40, ×100, ×1500 magnitude).

SEM analysis was used to examine the surface morphology of the hydrogels that were produced, as shown in Figure 4. All of these types of hydrogels typically had a 3D net-like structure made of clearly defined interconnected macropores, similar to other freeze-dried gel samples [41,42]. Generally, polysaccharides-based hydrogels have higher water contents, which made it easier for water to sublimate into larger pores following the freeze-drying process.

The presence of pores in the biomaterials is crucial because it enables an increase in oxygen concentration in the lesion, which is required for a number of cellular activities, including phagocytosis, mitosis and the release of growth factors, which are essential for damage repair [43].

After the addition of the silver nanoparticles, the morphology of the samples remained the same, with all of them exhibiting a highly porous structure with a lamellar shape highly beneficial for medical applications [43].

**Figure 5:**
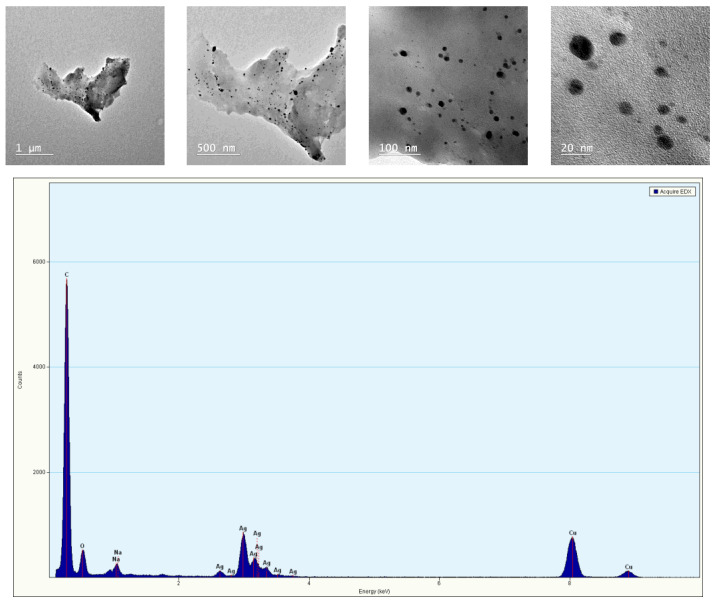
TEM images captured on SA1 sample and EDX spectrum.

TEM images are crucial because these provide information regarding the shape and dimensions distribution of the silver nanoparticles from the obtained samples (Figure 5).

TEM images collected on sample SA1 showed individual spherical silver nanoparticles, but also their agglomerated state. The dimensions ranged between 10 and 40 nm. The EDX spectra revealed the presence of a silver peak, confirming the inclusion of silver nanoparticles into the hydrogel crosslinked sample.

Thus, TEM images demonstrated the presence and the nanometric dimension of the inorganic component in the biopolymer matrix with the development of novel salecan-based nanocomposites.

### 3.2. Thermal Behavior of the New Antimicrobial Hydrogels

**Figure 6:**
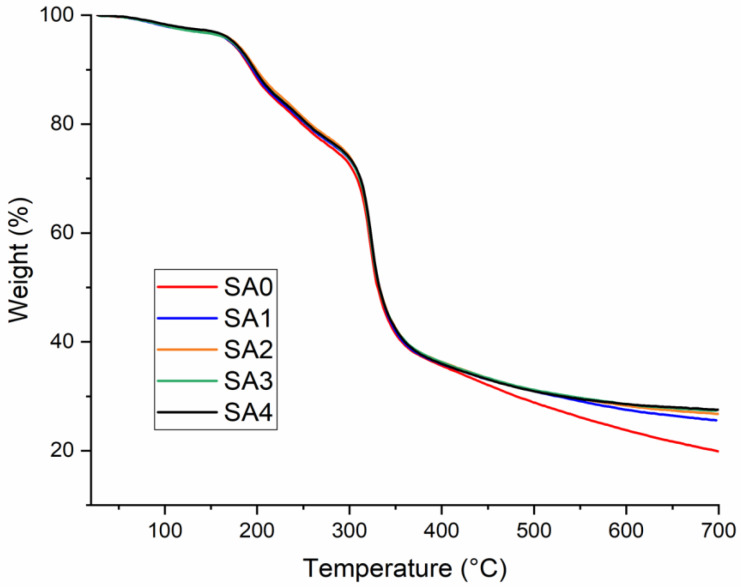
Thermal properties of the obtained samples.

The thermal stability of the new antimicrobial hydrogels was investigated using TGA analysis. The TGA obtained thermograms of the hydrogels based on salecan loaded with silver nanoparticles, presented in Figure 6, and these showed that all the samples exhibited several stages of weight loss. For all the obtained samples, the first stage, which ranged in temperature from 50 to 200 °C, caused a weight loss of around 10% and was linked to the evaporation of the absorbed water and mildly volatile substances [24]. The second (<50%) and third disintegration phases, which were discovered between 250 and 350 °C and 350 and 700 °C, respectively, showed the highest weight losses. These steps were associated with a complex process of breakage and breakdown that involved the rupturing of the strong covalent crosslinks, as well as the fragmentation of the polysaccharide backbone, including the saccharide ring’s C-O and C-C bonds [34,44].

In Table 2, the thermal properties of the obtained samples are summarized.

In Table 2, it can be observed that the addition of the silver nanoparticles does not significantly influence the thermal stability of the obtained hydrogels.

### 3.3. The Behavior of Crosslinked Hydrogels in Wet Conditions

Following the esterification reaction, covalent bonds resulted between the biopolymer networks by means of the green cross-linking agent, namely, citric acid, and strong hydrogels that are stable in a wet environment were obtained. Analogous citric acid cross-linked hydrogels were previously obtained by other growth groups which confirmed that the citric acid esterification cross-linking method is an efficient method of obtaining stable cross-linked hydrogels in a wet environment [29,30,31,37,38].

The resulting hydrogels were washed of unreacted products and, following UV testing, the degree of cross-linking of the salecan was calculated. Cross-linking degrees of ~ 95% were obtained regardless of the salecan hydrogel tested; thus, no changes were observed after the addition of inorganic nanoparticles, probably due to the very small amount of filler which does not have the ability to influence the formation of the cross-linked polysaccharide network. Great stability of the hydrogels in deionized water was noted, the samples being taken over 4 days. After 2 days, the samples already do not elute salecan, as revealed by UV tests by the disappearance of the characteristic salecan peak at 490 nm. However, as an assurance, the samples were kept under the same conditions and tested for the release of unreacted salecan.

In order to examine hydrogel’s stability in wet conditions, the samples were placed in different pH media. It is worth mentioning that the samples did not change their shape due to fluid retention and showed an advanced stability of the cross-linked form for 24 h. Thus, relatively high equilibrium degrees of swelling were calculated, with samples retaining up to ~650–750% of fluid relative to their dry mass. Such behavior is due the hydrophilic nature of the synthesized hydrogels abundant in OH and COOH groups and it is of great use when these materials are intended for use in biomedical applications, being ideal candidates for the ultimate retention of bioactive substances.

Thus, the samples showed degrees of swelling of ~650% in acid medium, at neutral pH having values of ~715%, the samples being stable and undegraded after more than one month in the mentioned media without differences between the synthesized compositions. On the other hand, at a basic pH, the samples behaved differently, i.e., after reaching a maximum swelling at 24 h of ~750%, the samples started to degrade progressively so that, after 72 h, the degradation was complete in the basic medium for all the synthesized samples without differences between them. These variations in swelling/degradation behavior are due to the protonation of OH groups at acidic pH; at a basic pH, instead, COOH groups undergo deprotonation to the anion carboxyl group, and in this case, repulsive forces intervene with consequences on the degradation of the samples [45].

These data can be useful for different applications that require high pH stability of the skin, such as wound dressing or degradation in basic bioactive release environments such as the gastrointestinal tract.

### 3.4. Mechanical Tests

**Figure 7:**
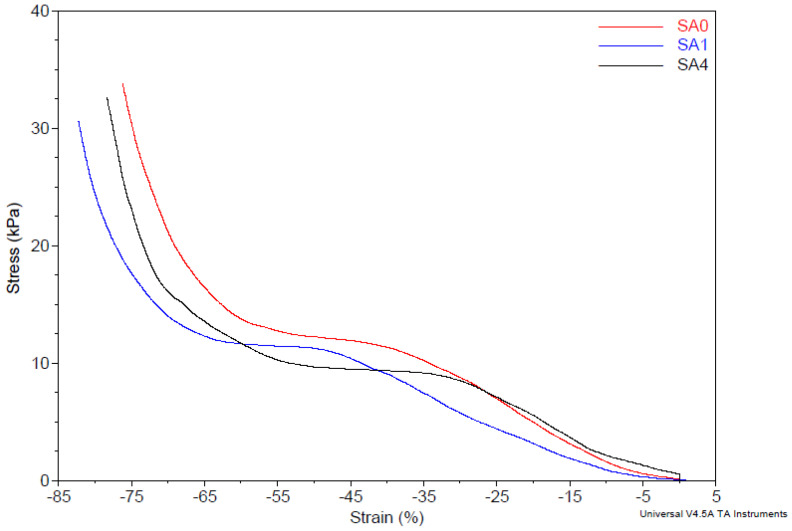
Stress–strain curves of the equilibrium-swelled hydrogel samples.

The strain–stress curves of the polysaccharide crosslinked hydrogels under compression are depicted in Figure 7. The compressive modulus increased with the presence of silver content in the hydrogel matrix; these results suggested that the introduction of silver nanoparticles could effectively improve the mechanical properties of the nanocomposite hydrogels, which is attributed to the interaction of the polymer chains with the silver nanoparticles. This interaction involved a local heterogeneity with respect to polymer segmental mobility, and therefore led to molecular stiffening and the consequent rearrangement of the polymer structure [46]. In conclusion, by varying the content of silver nanoparticles, we developed a new nanocomposite hydrogel system that has improved mechanical properties.

### 3.5. Antimicrobial Activity

**Figure 8:**
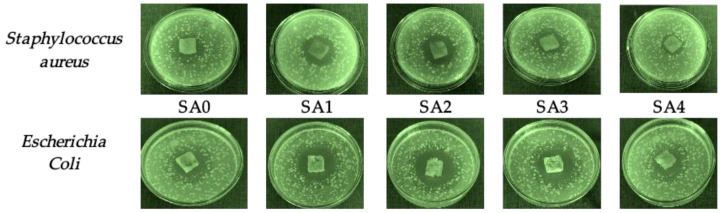
Antimicrobial activity against *Staphylococcus aureus* and *Escherichia coli* of the obtained hydrogels.

The antibacterial activity represents an important feature for several hydrogels used as bioactive wound dressings [40]. The antimicrobial activity of Ag NPs is generally recognized. It has been reported that Ag NPs is efficient against two strains: *Staphylococcus aureus* (Gram-positive bacteria) and *Escherichia coli* (Gram-negative bacteria) [40,47]. For all the obtained salecan-based hydrogels, the antimicrobial activity was evaluated (Figure 8).

In Figure 8, it can be observed that all the samples presented antibacterial activity against both of the tested strains. The sample SA0, without silver nanoparticles, presented a detectable zone of inhibition against the *Staphylococcus aureus* and *Escherichia coli* duo to its citric acid content, which is known to present antimicrobial activity [40].

The results presented in Table 3 revealed that all hydrogels loaded with silver nanoparticles presented higher microbiological activity and inhibition zones than SA0 for any of the bacteria tested.

For the obtained hydrogels, the zone of inhibition varied from 18 to 30 mm. The largest inhibition zone was presented by the sample SA1, which had the highest concentration of silver nanoparticles for both of the tested strains. Additionally, it can be observed that the zone of inhibition against both bacteria was increased by raising the amount of silver nanoparticles in the obtained hydrogels. Additionally, the difference between the two values in the degree of inhibition for the two types of bacteria tested after the addition of silver nanoparticles can be found using many different factors, such as the initial bacterial count in the suspension, the sensitivity of the specific strain and the culture medium type. The antibacterial activity of the silver nanoparticles is very strain-specific, and even different strains of the same species show varied sensitivity, according to numerous research studies [48]. Additionally, numerous studies have demonstrated that *Staphylococcus aureus* and *Escherichia coli* have differing sensitivities to silver nanoparticles, albeit, these results differed since, in some studies, *Escherichia coli* was more sensitive than *Staphylococcus aureus*, whereas in others, it was the other way around [48,49]. Other silver nanoparticles-loaded hydrogels proved the inhibitory effect of the selected bacterial strains, such as nanocomposite hydrogels based on alginate/gelatin [50], alginate [51], gelatin/polyvinyl-alcohol [52], carboxymethylcellulose [53] and poly (methacrylic acid-acrylamide) hydrogel [54].

Although it is speculated that the distinct cell wall architectures of Gram-positive and Gram-negative bacteria may result in differing sensitivities, the precise mechanism causing this phenomenon has not yet been identified [55]. In this research study, *Escherichia coli* was shown to be more resistant compared to *Staphylococcus aureus* for the majority of the samples; in addition, Nešović et al. [29] reported the same in their research works.

The obtained results reveal that our composites hydrogels can be considered as a good antibacterial biomaterial for wound dressing applications.

## 4. Conclusions

In this research work, novel bioactive nanocomposite hydrogels were successfully synthesized. Strong covalent bonds were achieved through the esterification of salecan polysaccharide with citric acid, which assured enhanced stability in wet conditions. The variation of the ratio between the hydrogel components did not significantly influence the morpho-structural properties of the final nanocomposites, according to FTIR and ESEM microscopy analyses. TEM images showed the presence and nanometric dimensions of the inorganic component in the biopolymer matrix, demonstrating the development of novel salecan-based nanocomposites. The improved mechanical properties of the nanocomposite hydrogel system were determined to be a consequence of restricted polymer segmental mobility in the presence of silver nanoparticles. Most importantly, the crosslinked hydrogels presented great inhibitory activity against *S. aureus* (Gram-positive bacteria) and *E. Coli* (Gram-negative bacteria) due to the recognized antibacterial effect of citric acid. Due the further incorporation of silver nanoparticles, the inhibition areas were increased for both bacterial strains, denoting a synergistically enhanced behavior for the obtained nanocomposites. Therefore, the novel bioactive crosslinked hydrogels could be potential biomaterials in medical field of wound dressing applications.

## Figures and Tables

**Table 1:** Composition of the obtained hydrogels.

Sample	Salecan [g]	Citric Acid Solution 5% [mL]	Silver Nanoparticles [g]
SA0	0.75	10	-
SA1	0.75	9	0.01
SA2	0.75	9.5	0.005
SA3	0.75	9.75	0.0025
SA4	0.75	9.9	0.001

**Table 2:** Thermogravimetric properties of the obtained hydrogels.

Sample	T_10%_ [°C]	T_50%_ [°C]	Thermal Degradation Step, /DTG T_max%_ [°C]	Residual Mass [%]
SA0	192	330	324	20
SA1	195	332	325	26
SA2	200	333	322	27
SA3	197	332	324	27
SA4	197	332	323	28

**Table 3:** Evaluation of the antimicrobial activity of the hydrogels loaded with silver nanoparticles.

Sample	*Staphylococcus aureus*		*Escherichia coli*	
	Inhibition Zone (mm)	STD	Inhibition Zone (mm)	STD
SA0	18	0.1	18	0.1
SA1	30	0.2	24	0.1
SA2	29	0.1	23	0.1
SA3	25	0.1	23	0.2
SA4	20	0.2	22	0.1
